# Study Protocol: Social Capital as a Resource for the Planning and Design of Socially Sustainable and Health Promoting Neighborhoods— A Mixed Method Study

**DOI:** 10.3389/fpubh.2020.581078

**Published:** 2020-10-19

**Authors:** Ailiana Santosa, Nawi Ng, Liv Zetterberg, Malin Eriksson

**Affiliations:** ^1^School of Public Health and Community Medicine, Institution of Medicine, Sahlgrenska Academy, University of Gothenburg, Gothenburg, Sweden; ^2^Department of Epidemiology and Global Health, Faculty of Medicine, Umeå University, Umeå, Sweden; ^3^Department of Social Work, Umeå University, Umeå, Sweden

**Keywords:** social capital, sustainability, health promotion, neighborhood, mixed method approach

## Abstract

**Introduction:** Promoting inclusive, safe, resilient, and sustainable communities is one of the 17 Sustainable Development Goals ratified in 2015 by 193 UN member states, not least in Sweden. Social sustainability involves preserving particular societal values (e.g., local identity) as well as developing values (e.g., social cohesion) that are perceived as needed. Socially sustainable development also implies promoting integration and preventing segregation. Social capital is one important indicator to measure how socially sustainable an area is. This project aims to explore how social capital can be used as a conceptual tool in developing housing policy for social sustainability in Umeå Municipality.

**Methods:** The three sub-studies in this project combine quantitative and qualitative methods. We will conduct a review of the municipality's documents to understand how the ideas of social sustainability have influenced political declarations and implemented social and housing policies and interventions during the period 2006–2020. The quantitative study includes a longitudinal follow-up to the 2006 survey's respondents to assess the longitudinal impacts of neighborhood social capital on health and well-being; as well as a new repeated cross-sectional survey to investigate how social capital has changed in local neighborhoods from 2006 to 2020. The qualitative study includes case studies in neighborhoods with different social capital dynamics to understand how different resident sub-groups perceive their neighborhoods and how implemented social and housing policies have influenced the social capital dynamics and responded to the needs of different sub-groups. The project is run in close collaboration with the Commission for a Socially Sustainable Umeå.

**Discussions:** This project will create new and unique perspectives on long-term structural changes of relevance for a socially sustainable housing policy; knowledge that is highly valuable for continuous municipal planning; and will outline recommendations to guide local housing policies for social sustainable neighborhoods in Umeå Municipality.

**Ethics:** This study has been assessed and approved by the Swedish Ethics Review Authority (Dnr: 2019-04395; Dnr: 2020-00160; Dnr 2020-02757).

**Dissemination:** The dissemination goals of this project are (1) sustained engagement of key stakeholders throughout the project and (2) dissemination of the research findings through popular science, conferences, and scientific papers.

## Introduction

Promoting inclusive, safe, resilient, and sustainable communities is one of the 17 Sustainable Development Goals (SDGs) ratified in 2015 by 193 UN member states including Sweden. This implies creating communities with equal opportunities for all and access to basic services, housing, and transportation (1). Sweden does well in housing, heating, waste management, and access to green areas but still faces problems when it comes to perceived safety in public spaces (not least for women), segregation in local neighborhoods (with 61 neighborhoods classified as socially vulnerable) and accessibility of services (not least for people with disabilities) (2). These challenges reflect issues in social values. Social sustainability is stated as being of equal importance to economic and environmental sustainability in order to reach the SDGs (1) but the term is still not clearly defined (3). According to Statistics Sweden, there are few available indicators on measuring Sweden's performance on the SDG 11, i.e., how to make cities and communities inclusive, safe, resilient, and sustainable (4). Ström et al. conclude that social sustainability allows many different interpretations but involves preserving particular societal values (e.g., local identity) as well as development of values (e.g., social cohesion) that are perceived as needed (3). The Swedish National Board of Housing, Building, and Planning additionally underlines that socially sustainable development implies promoting integration and preventing segregation (5). Thus, social sustainability clearly relates to the concept of social capital.

Social capital has been widely used in policy and research over the last decades, including within developmental studies (6) and health research (7, 8). Despite its multidimensional meanings, the theoretical and empirical development of the concept has led to established operationalization of its measurements (7). Social capital involves “social networks, the reciprocities that arise from them and the value of these for achieving mutual goals” (9), and is conceptualized as both an individual and a collective feature. Social capital, conceptualized as characterizing a local area, emanates from the work of the political scientist Robert Putnam (10, 11). According to Putnam, a community high in social capital is characterized by the existence of dense and strong social networks, high involvement in these networks, and strong norms of reciprocity and generalized trust between people. Thus, social capital can be useful as a conceptual tool in local policies for social sustainability (10, 11).

Putnam studied the effects of social capital and found that states in the US that were high in social capital did better in terms of education, safety, economy, democracy, and health (11). There is growing evidence for a positive association between living in a place with high social capital and health, at least for some populational groups (12). However, in our previous social capital survey in Umeå Municipality, we found an association between living in a high social capital neighborhood and good self-rated health for women but not for men (13). Similar gendered patterns have been found in studies from the UK (14) and Australia (15). These studies indicate that women may benefit more from living in high social capital neighborhoods than men. In order for social capital to become a gender equal resource in the planning and design of socially sustainable and health promoting neighborhoods, the gender differences in the effects of place-specific social capital and health needs further exploration.

One major limitation in most studies about social capital and health is its cross-sectional nature, making it difficult to rule out causal effects of social capital on health (16). In these studies, reverse causality could not be ruled out; healthy people may be more likely to be civically engaged and trusting and may also be more likely to live in areas where a majority are the same. Longitudinal studies can ascertain causal effects of neighborhood social capital on health but are scarce, hence urgently needed. Moreover, the question of how social capital develops in local areas over time and how it may be intentionally generated, is still unexplored (17). This project will contribute to filling in these gaps in knowledge.

Social capital, conceptualized as a collective, place-specific feature will be used as our theoretical framework since it clearly relates to the concept of social sustainability. Macintyre et al. state that neighborhood environments may influence health through the material infrastructure as well as through the *collective social functioning* of the neighborhood (18). The latter aspect relates to social capital and social sustainability. There are different hypotheses about the link between place-specific social capital and health. Kawachi et al. stress that socially cohesive neighborhoods are better in uniting for the good of their community, which may be positive for residents' health (19). Further, social capital can influence health through faster and wider diffusion of healthy norms and information, since health information and norms spread more effectively in areas where people trust and interact with each other (19). Thus, social capital is seen as a non-exclusive good, in that living in a high social capital neighborhood may benefit even individuals with poor social connections (11).

There is still a lack of theoretical and empirical support on how social capital can be generated intentionally. However, a literature review about the role of social capital in local development (20) found the following initiatives to be of importance for generating social capital in local areas: investments in the physical environment that facilitate social interactions and safety; planning and designing attractive meeting places and green areas; efforts to improve an area's reputation; organizing community activities that are perceived as meaningful and attractive; and promoting local associations with a clear inclusive strategy. This project will contribute further theoretical and empirical support on how to generate social capital and thus social sustainability in local neighborhoods.

### Aims and Objectives

The overall aim of this project is to explore how social capital can be used as a conceptual tool in developing housing policy for social sustainability at municipality level. The specific objectives of this project are: (1) to investigate the dynamics of social capital in local neighborhoods over time, as well as to explore how housing and social policies and sociodemographic composition are associated with neighborhood social capital over time; (2) to explore how local residents perceive the development of social capital and the impacts of housing and social policies in their neighborhoods; and (3) to analyze the cross-sectional associations and longitudinal causal effects between neighborhood social capital and health, and whether these associations are gendered.

## Methods

### Study Context

This project emanates and further builds on our previous studies on social capital and health, conducted in the Umeå Municipality in northern Sweden (13, 21). The northern Sweden region ranks high in social progress with regards to basic human needs and foundations of well-being (22). Further, Umeå Municipality stands out as a municipality in Sweden with high levels of social capital (23). The overall vision for Umeå Municipality is to be a home for 200,000 inhabitants by 2050 (in 2017 it was 122,892). In order to fulfill this vision, rapid and extensive housing is needed. As decided, the annual number of new houses should amount to 2000 over the coming years. Evidently, this growth implies challenges concerning social sustainability. The establishment of the Commission for a Socially Sustainable Umeå in 2017 is one strategy for meeting these challenges. In the strategic development goals for Umeå Municipality, social sustainability is clearly reflected in two out of the six goals, namely; “promoting public spaces and parks” and “ensuring social inclusion” (24). The Commission's task is to analyze differences in living conditions in Umeå Municipality between groups and geographic areas, as well as to provide concrete measures for socially sustainable development throughout the municipality. Our proposed project is closely connected to the work of the Commission since our research questions fit well within the Commission's overall aim. A “policy to practice” reference group is set up to facilitate the collaboration between the research group and the Commission in Umeå Municipality. This project is carried out in collaboration with the commission in Umeå Municipality. Thus, our research is “community based,” but does not adhere to the principles of community based *participatory* research, since our municipal collaborators will not be equally involved in the research with regards to participation, influence, and control (25).

Findings from our previous survey among 5,768 residents in Umeå Municipality in 2006/2007 showed that social capital was distributed unequally between neighborhoods within the municipality (13). Living in a high social capital neighborhood increased the odds for good self-rated health among women while not among men (26). Later, a qualitative focus group study was conducted to explore people's perceptions of health promoting neighborhoods (26). The results showed that neighborhood social capital, together with other elements in the living environment, clearly influence people's perceived health, but the results did not confirm that social capital is more important for women than for men. Another study examined the significance of neighborhood social capital on children's health and found that living in a high social capital neighborhood was protective of child (0–12) injuries among girls while not for boys (21). In addition, a qualitative Grounded Theory and Photovoice study has been conducted to explore children's perspectives on health promoting living environments (27).

### Study Design

We designed the current project as a mixed method study, combining a desk review of official documents with quantitative and qualitative studies. The quantitative study includes a longitudinal follow-up to the 2006 survey's respondents, to assess the longitudinal impacts of neighborhood social capital on health and well-being and a new repeated cross-sectional survey to investigate how the level of social capital has changed in local neighborhoods from 2006 to 2020. The qualitative study includes case studies in neighborhoods with different social capital dynamics over time. It will generate data about how different resident sub-groups perceive their neighborhoods and how implemented social and housing policies have influenced the social capital dynamics and responded to the needs of different sub-groups. The results of both the quantitative and the qualitative studies will be compared between men and women; this knowledge can be used to plan and design for gender equal policies to influence social capital in local neighborhoods.

#### Desk Review

We will conduct a review of municipality strategic planning documents, political decisions, policy reports, and monitoring documents during the period 2006–2020. The review will generate organizational level data on which social and housing policies and interventions have been implemented in different neighborhoods, as well as on how the ideas of social sustainability have influenced political declarations and implemented initiatives during the same period. We are also interested in documents and reports concerning the political and administrative decision process regarding these issues, as well as documents that form the basis for those decisions. All the municipal documents are publicly available according to the Swedish principle of public access to official records. To find relevant documents, we have started to search Umeå Municipality's website.

#### Quantitative Study

Both the longitudinal follow-up and the repeated cross-sectional surveys are conducted by Statistics Sweden which provides the sampling frame for the selection of the study participants, conducts the survey during spring 2020, and provides the survey datasets to the researcher. We use the same protocol and instruments as were used in the baseline 2006/2007 survey. The original questionnaire was developed based on a review of existing international instruments and adapted to a northern Sweden context (13). We measured neighborhood social capital based on people's perceptions about their neighborhoods; whether neighbors talk to each other, help each other, are expected to be involved in issues concerning the neighborhood, and care for each other. Responses from the survey participants were used to derive the neighborhood social capital score for each of these neighborhoods, by aggregating the individual-level composite scores at the neighborhood-level (high composite scores represent neighborhoods with high social capital, and vice versa).

In the current survey, we include questions on socioeconomic factors, perceptions about living area, civic and political engagement, reciprocity and trust, social networks, social support, self-rated health, and health-related quality of life measured using the RAND-36 instrument. We link the survey's data to the national drug prescription and hospitalization register to get more objective health measures and to the Swedish Longitudinal Integrated Database for Health Insurance and Labor Market (LISA data) to obtain sociodemographic and economic variables at individual and neighborhood level.

In the current project we will utilize the same neighborhood division as well as the same measurement of neighborhood social capital as in the baseline study of 2006/2007. Neighborhoods are constructed based on postcode sectors in the geographical areas officially recognized by the municipality as well as by people in general based on local knowledge (i.e., by defined neighborhood and village names). Several geographically close postcode sectors are merged to fit the geographical borders of the larger neighborhood areas. In the 2020 survey the neighborhood division has slightly changed (with the amalgamation of a few postcode sectors and the construction of newly developed residential areas, hence new postcode sectors), resulting in 46 defined neighborhoods.

For the repeated cross-sectional survey in 2020, we used the sampling frame of 100,021 individuals aged 18–84, living in Umeå Municipality. Based on the proportion of people with poor health which ranged from 6.2 to 13.2% in neighborhoods with low vs. high social capital in our survey in 2006, we estimated a sample size of 148 in each neighborhood, with an α (alpha) of 5% and maximum error of 3.9%. We assumed a response rate of 40%, resulting in a total number of 16,000 individuals aged 18–84 years old who lived in the 46 neighborhoods in Umeå Municipality being recruited into the cross-sectional study.

For the follow-up study, we recruited the 2006/2007 survey respondents who left their personal id-number voluntarily (*n* = 3,600 individuals, about 58% of all participants). At the end of 2019, 2028 individuals were still living in Umeå Municipality and were recruited to the study.

The survey questionnaire was sent in January 2020 via ordinary mail to the respondents' registered home addresses. The respondents participated by filling in the paper questionnaire or online questionnaire through a weblink. By the end of February 2020, we had obtained 37% of all the responses (33% in the cross-sectional survey and 65% in the follow up survey). We assume that the responses to the survey were not affected by the Covid pandemic, as there were only 14 Covid cases reported in Sweden by 29th February 2020 and none in the Västerbotten Region where Umeå Municipality is located. As more responses were sent back to Statistics Sweden during March-April 2020, we decided to conclude the surveys at the end of June 2020. We obtained a response rate of about 40% for the repeated cross-sectional survey and 70% for the follow-up study. The sample size ranges from 14 individuals in the smallest neighborhood to 402 individuals in the largest neighborhood.

To further increase the response rate in the follow-up study, we are contacting the non-responders by telephone with a request to take part in a short telephone survey. A shortened version of the survey has been constructed for this purpose. Some of the questions in the survey needed to be adjusted to the current situation with the coronavirus pandemic. We have also been granted the ethical permission to ask some Covid-19-related questions. These questions include questions on health, whether the respondent has given and/or received help from a neighbor during the pandemic, perceptions about any changes (less/same/more) as to whether neighbors talk to each other, help each other, are expected to be involved in issues concerning the neighborhood, and care for each other.

#### Qualitative Study

##### In-depth interviews

Qualitative interviews with different stakeholders will be conducted concurrently and after the quantitative data collection is done. We are currently in the phase of recruiting respondents and have been conducting some initial interviews. First, we plan to interview strategic municipal planners and other relevant actors in Umeå Municipality to obtain the official version of social development within the municipality over time. Thereafter, we will interview key community members in some selected neighborhoods to explore how residents perceive their neighborhood and its development over time.

The first interviews will give us general knowledge on policies and interventions in the municipality regarding decisions, and interventions connected to social and housing issues during 2006–2020. We are also interested in how ideas of social sustainability are articulated and turned into practice. For these first interviews, we will recruit respondents who hold strategic positions in the municipality; roles that have granted them an overview of the organization and its decision-making. Sampling of respondents will be based on snowball sampling. We will start interviewing the municipality's strategic development coordinator and the information gained from this interview will be used to identify further relevant respondents. In the interviews, relevant documents for the desk review can also be identified and respondents can in turn be identified in the documents. Thus, these interviews will follow an emergent design. Four interviews have been conducted so far and we foresee a need for 6–10 stakeholder interviews in total.

The collaboration with Umeå Municipality and the Commission for a Socially Sustainable Umeå is in many respects an asset, both for data collection and result dissemination; but it also poses some challenges. Since the people that are involved in the Commission often are those who have the most knowledge about aspects that we are interested in, some municipal officials are both project partners and respondents. This places high demands on the researcher to be aware of any bias, keep a critical perspective on the respondents' stories, and to also feel free to make any criticism when the results are to be reported.

Based on the mapping in the quantitative study, we will strategically select four neighborhoods for qualitative case studies. They will be neighborhoods with different social capital dynamics that have (a) dropped in social capital rank; (b) gained in social capital rank; (c) remained the same in social capital rank; and (d) been newly established. More detailed documents and papers will be collected for these neighborhoods in the desk review. To get an in-depth knowledge of the chosen neighborhoods we will then recruit respondents in Umeå Municipality with a detailed knowledge of specific decisions or interventions that have been carried out in these neighborhoods. In the four chosen neighborhoods, interviews with purposely selected community members will be conducted. These qualitative case studies will explore how residents perceive their neighborhood and its development over time. We will start by identifying people involved in locally based associations or networks in each neighborhood. Thereafter we will aim to find respondents in a wide range of positions, with different social networks and who have knowledge of the situations of different groups. One could argue that a heterogeneous sample is theoretically motivated (28), since the latter sampling will be based on ideas gained from the previous interviews. Interviews with 3–4 key persons will focus on perceptions and experiences of the social climate; the composition of people in the neighborhood and whether people that are dissimilar mix with each other; reflections about how this has changed over time; as well as opinions and experiences of housing and social policies that have been implemented in the neighborhoods. Interviews will be recorded and transcribed.

##### Focus group discussions

We will conduct focus group discussions (FGDs) with purposely selected residents in the same four neighborhoods. FGDs will cover some key issues identified from the interviews and will enable comparisons of attitudes and experiences between different resident sub-groups. Based on interview data, we will construct statements about the social climate in the selected neighborhoods to be used in the FGDs. In order to grasp a broad view of experiences and perceptions, we plan to conduct FGDs with people in different age groups, i.e., retirees (65+), people of working age (30–65), as well as children and young people (12–15). FGDs will be sex-stratified to further explore gender differences in the perceptions about the living environment. The decision to stratify groups by age and gender is motivated both theoretically—since the level and the effects of social capital vary between these groups—and by concerns about power and status in the group dynamics (29). The recruitment will go through different organizations, clubs/communities, and citizen groups, such as religious communities, sports clubs, staff at schools or leisure centers, or NGOs. This will help us in reaching participants with access to different networks, with different interests and in different life circumstances. One of the research team members will act as the FGD facilitator and moderate the discussion. We plan to conduct about six FGDs (with 4–6 participants) in each neighborhood.

The focus groups with children (12–15 years) will be preceded by photovoice activities, i.e., they will be allowed to borrow simple digital cameras to photograph places in their residential area. The photos will then be used as a basis in the group discussions where the children will be asked to present and discuss their photos. Photovoice sessions will be carried out in collaboration with *Kulturverket*—a municipal agent working with creative workshops for children—and local schools, according to a model that was developed and tested by the research team in a previous study. The focus groups will be conducted in the school during regular school hours. The FGDs will be recorded and transcribed. Focus groups with children require adapted specific tools and careful ethical considerations. In this project we utilize and build further on an approach that was developed and tested in our previous studies involving children in the same research setting (27).

### Sub-Studies and Data Analysis

#### Sub-Study 1: Study on the Dynamics of Neighborhood Social Capital in a Swedish Municipality

This sub-study will be based on mixed method design, combining quantitative (survey) and qualitative (interviews and document reviews) data. In this sub-study, the research questions will focus on: (i) how the levels of neighborhood social capital have changed in Umeå Municipality over a period of 12 years and the neighborhood-level factors that may affect the changes in social capital; (ii) whether implemented social and housing policies and interventions as well as changes in socio demographic composition in the neighborhood explain changes in neighborhood social capital level; and (iii) whether interventions in new and existing neighborhoods have been planned in line with guidelines and principles for social sustainability.

We will rank neighborhoods based on their social capital scores and will compare each neighborhood's ranking in the baseline and repeated survey using the non-parametric Wilcoxon Signed Rank test. The aggregated data of sociodemographic and socioeconomic profiles of the residential areas between 2006 and 2017 that are constructed by Statistics Sweden will be used for examining whether changes in those sociodemographic and socioeconomic profiles affect each neighborhood's social capital level.

#### Sub-Study 2: Qualitative Study on Local Residents' Perceptions on the Development of Social Capital at Neighborhood Level

This qualitative study will follow up on the findings from sub-study 1. The following research questions will be explored in this sub-study: (i) Are the survey results on the dynamics of neighborhood social capital confirmed by people's subjective perceptions about their neighborhoods? and (ii) How do residents perceive changes in socio demographic composition as well as housing and social policy in these neighborhoods?

We will analyze the interviews and FGD data using Constructivist Grounded Theory and Situational Analysis. Next, we will compare the qualitative data against the survey data and document reviews from sub-study 1. We will explore how the results from the social capital mapping and the planning documents are reflected in residents' own perceptions of their neighborhoods. This will give an in-depth understanding of how changes in socio demographic composition as well as implemented social and housing policies influence people's perceptions about their living environment and its influence on their health.

#### Sub-Study 3: Estimation of Causal Effects of Neighborhood Social Capital on Individuals' Health

This sub-study builds on the quantitative data to answer the following research questions: (i) what are the effects of living in neighborhoods with low- vs. high-levels of social capital on health using observational cross-sectional data? and (ii) how long-term exposure to different levels of neighborhood social capital, for individuals who stayed in the same neighborhood and those who moved between neighborhoods, affected their health, after controlling for individual-level socioeconomic and health determinants as well as neighborhood-level socioeconomic and demographic changes?

We will estimate the “treatment effect,” which is based on the counterfactual potential outcome approach to model causal effects using observational data. We will compare individuals in neighborhoods with high (“treatment” group) vs. low (“control” group) social capital. Further, we estimate the causal effect of the treatment on the health outcomes as the difference between the two potential outcomes in the two groups. Using the follow-up data, we will estimate the difference-in-difference (DID) in health outcomes between the groups over time to obtain an appropriate counterfactual to estimate a causal effect of intervention. The DID estimation is performed using an interaction term between time (baseline vs. follow up) and treatment group variables in the multilevel logistic regression model. The multilevel model will be employed to assess the fixed effect (the effect of neighborhood social capital on health) and random effect (variation in health outcomes that could be attributed to neighborhood level). We will stratify the analysis by sex to test if the association between neighborhood social capital and health are gendered.

## Ethics and Dissemination

### Confidentiality

Participation in this project is completely voluntary and all data will be treated confidentially. All participants will receive thorough information on how data is used and managed. Personal data within the repeated cross-sectional study is handled by Statistics Sweden. While for the persons included in the follow-up study, personal data will be handled by the research team. The research team is aware of the risk of privacy infringement with this procedure, especially when questionnaire data from two measurement occasions is to be linked with health register data over time. Therefore, the data file will be de-identified by removing the social security numbers and replacing them with serial numbers before starting analysis. Thus, all analyses will be performed on unidentified data. The results will only be presented at an aggregated level.

Regarding the qualitative sub-study, the focus of the interviews, focus groups and photovoice activities is on perceptions of the residential area, and therefore the participants will not be asked to answer any individual questions of a sensitive nature, such as health, sexual orientation, or political opinion. The research team's experience from previous research is that questions about the importance of the housing area for self-perceived health are of a much less sensitive nature than, for example, questions about lifestyle and health-related behaviors.

When it comes to using children's and young people's photographs, we are aware of the risk of privacy infringement if photos are taken of people who have not been asked and if photos are disseminated in social media. However, the research team has experience in managing these risks from previous research (27). Children will be asked to take pictures only of places, not of people. If photos contain pictures of people who can be identified, these images are sorted out and not used. Photographs are taken only with a borrowed digital camera and not with the research subjects' own mobile phones, which reduces the risk of images being shared on social media. The specific ethical considerations that need to be taken concerning involving children in research has been assessed and approved by the Swedish Ethical Review Authority.

### Dissemination

This project will create new and unique perspectives on long-term structural changes of relevance for a socially sustainable housing policy; knowledge that is highly valuable for continuous municipal planning. Based on the results of the project, we will outline recommendations to guide local housing policies for socially sustainable neighborhoods in Umeå Municipality. These recommendations will be discussed in the reference groups and presented to local politicians within the work related to the Commission for a Socially Sustainable Umeå. In summary, this project can strategically contribute to the Commission's tasks. The constructed social capital questionnaire from this project can be used and evaluated as one way of measuring social sustainability in Umeå Municipality where local indicators for how to measure social sustainability in the municipality are yet to be developed. The results of our research can be used to promote scientific evidence-based policymaking in promoting a socially sustainable development in Umeå Municipality.

The dissemination goals of this project are (1) sustained engagement of key stakeholders throughout the project and (2) dissemination of the research findings. Several activities are planned such as meetings between the reference group and the involved researchers on a regular basis throughout the project in order to achieve mutual discussions about project design, data collection, and preliminary results. Joint workshops and seminars will be organized to disseminate the preliminary results of the project. This will ensure that the project results will be spread and discussed within the municipal organization as well as with other collaborating actors such as property owners. The findings from the projects are intended to be disseminated through international peer-reviewed scientific Open Access journals, international and national conferences, and seminars.

## Discussions

### Anticipated Results

The projected growth in the number of Umeå inhabitants by 2050 implies there will be challenges around social sustainability. The establishment of the Commission for a Socially Sustainable Umeå in 2017 to meet these challenges has to be applauded. The Commission is assigned to “analyse differences in living conditions in the municipality of Umeå between groups and geographical areas and propose concrete measures for socially sustainable development throughout Umeå Municipality.” Yet, local indicators for measuring and monitoring social sustainability in Umeå Municipality are yet to be developed. In this project, our constructed social capital indicator will be used and evaluated as one way of measuring social sustainability in Umeå Municipality. The indicator allows for comparisons between neighborhoods as well as between different resident groups. The planned sub-studies in this project will also contribute to analyses of differences in living conditions between neighborhoods as well as different population groups.

This project will have several key areas of impact. First, this project can provide new and unique perspectives on long-term structural changes of relevance for socially sustainable housing policy. Research can benefit participants by giving them knowledge about those housing environment factors that have a positive impact on the health of different social groups, and which can therefore be taken into account by municipal social planning for a socially sustainable housing construction. Second, since the project is carried out in close collaboration with the Comprehensive Planning Unit at Umeå Municipality, the results of this project can be used to promote scientific, evidence-based policy-making in promoting socially sustainable development in Umeå Municipality; it will outline the recommendations to guide local housing policies for socially sustainable neighborhoods. These recommendations will be discussed in the reference groups and presented to local politicians within the work related to the Commission for a Socially Sustainable Umeå. Therefore, this project can contribute strategically to the Commission's tasks. Even though we do not adhere to the principles of community based participatory research in this project, we expect that the results of this project should be used to plan interventions on how to promote socially sustainable and health promotive neighborhoods. Therefore, we foresee future research projects to be planned, designed, and carried out in close collaboration between researchers and the municipality, i.e., through community based participatory research.

As the European region with the highest social progress index, the Northern part of Sweden, where Umeå Municipality is located (22), plays an important role as the role model for other regions in Sweden and internationally in formulating their development strategies. The lessons generated in our project in Umeå Municipality, which is one of the fastest growing European cities, could therefore inform the development of a socially sustainable housing policy in other municipalities in Sweden, as well as communities globally.

There are clear links between the ideas about the importance of collective, place specific, social capital, and community development. The main purpose of community development (health-promotion) is to support community capacity to improve the foundation for a flourishing community. The ideas behind collective social capital offers an understanding of community-level determinants of health and social sustainability, with its focus on collective identities and collective action. Thus, mobilizing social capital in local communities could be seen as a key goal for the design of health promoting and socially sustainable neighborhoods. The community development literature will be used to discuss and understand the results in this project.

### Limitations

A few methodological challenges to be anticipated in the quantitative study include attrition and the representativeness of the individuals in the follow-up survey, the small sample size in small neighborhoods in the cross-sectional survey, and the change in neighborhood borders over time. Only 58% of the respondents in the 2006/2007 survey left their personal number voluntarily, and among them, only 56% were still living in Umeå Municipality by the end of December 2019. The resulting the follow-up study might be selective and not representative of the population of Umeå Municipality. Yet, as longitudinal data on social capital is scarce, the follow-up survey could contribute to an understanding of the dynamic of neighborhood social capital as perceived by the individual respondents. Any comparison of the findings for 2006 and 2020 needs to take into account the 2020 neighborhood border changes, as new living areas were established and merged into some of the existing neighborhoods. Even though the response rate of 40% in the repeated cross-sectional survey could be considered to be low, it is not less than the response rate in many other surveys in Sweden. The latest public health survey in Sweden was conducted in 2018 and reached a response rate of 42% (30).

The research project covers the period from 2006 to 2020. One methodological challenge will be to find respondents for the qualitative studies who have an overview and knowledge about such a long time period. This will probably be less of a problem for the first general stakeholder interviews and the community leaders than the other stakeholder interviews, as respondents with strategic roles in the municipality or in the neighborhoods have usually been in the organizations for a longer time. Information from the interviews and from the desk review will be used together to complement each other to give a full picture. For the focus group it is not necessary that all participants are long-term residents. On the contrary it could be enriching to have participants with different perspectives.

The current situation with the Covid pandemic will possibly have an impact on the study—both in terms of data collection and the result of it. The survey was sent out before WHO confirmed the first death worldwide and Covid was not yet a public issue in Sweden. When dissemination was confirmed in some parts of Sweden, in mid-March, most survey answers were already sent in and for those answers we believe that the pandemic did not impact on the answers. In the telephone survey that started in mid-June, the questions were slightly adjusted to refer to the time before the pandemic. The conducted qualitative stakeholder interviews have been carried out through video call so far, due to the situation. Our assessment is that this has been a good solution for this kind of interview. Further qualitative data collection is planned to start this autumn. For interviews with community leaders, video call interviews are an option, since these are mostly people in some kind of professional or semi-professional situation, or in leading positions within organizations; thereby having experience of being in similar situations and with professional access to the required technical equipment. Regarding FGDs with residents in the selected neighborhoods, the current situation will be a challenge. It will be difficult to carry out group discussions through video call since one feature of the group discussion method is the interaction between the participants (31). Another potential challenge with online focus groups is the requirement of technical equipment for the participants, and there are also considerable ethical considerations (32). The focus group discussions, and the photovoice activities with children, may therefore have to be postponed. If the situation goes on for too long, this could possibly affect how we relate the survey results to the group discussions since the situation in the neighborhoods could change.

### Conclusions

We anticipate that our project outcomes will have an immediate practical implication and impact on supporting evidence-based policy-making processes in promoting socially sustainable development in Umeå Municipality. Our close collaboration with Umeå Municipality will ensure an alignment between our research and the municipality's agenda. More practically, our findings will offer unique perspectives on long-term structural changes of relevance for a socially sustainable housing policy. This knowledge can be used for the planning and implementation of gender-equal and socially sustainable housing policies in local neighborhoods.

## Ethics Statement

The studies involving human participants were reviewed and approved by the Swedish Ethics Review Authority (Dnr: 2019-04395; Dnr: 2020-00160; Dnr 2020-02757). The patients/participants provided their written informed consent to participate in this study.

## Author Contributions

All authors contributed significantly to the concept, design of the manuscript, and critically reviewed and provided feedback on the final revised version submitted for publication.

## Conflict of Interest

The authors declare that the research was conducted in the absence of any commercial or financial relationships that could be construed as a potential conflict of interest.
